# Iron Accumulation with Age, Oxidative Stress and Functional Decline

**DOI:** 10.1371/journal.pone.0002865

**Published:** 2008-08-06

**Authors:** Jinze Xu, Mitchell D. Knutson, Christy S. Carter, Christiaan Leeuwenburgh

**Affiliations:** 1 Department of Aging and Geriatrics, Division of Biology of Aging, Genomics and Biomarkers Core of The Institute on Aging, University of Florida, Gainesville, Florida, United States of America; 2 Food Science and Human Nutrition Department, University of Florida, Gainesville, Florida, United States of America; 3 Gainesville VA Geriatric Research, Education and Clinical Center (GRECC), Gainesville, Florida, United States of America; Universidade de Brasília, Brazil

## Abstract

Identification of biological mediators in sarcopenia is pertinent to the development of targeted interventions to alleviate this condition. Iron is recognized as a potent pro-oxidant and a catalyst for the formation of reactive oxygen species in biological systems. It is well accepted that iron accumulates with senescence in several organs, but little is known about iron accumulation in muscle and how it may affect muscle function. In addition, it is unclear if interventions which reduced age-related loss of muscle quality, such as calorie restriction, impact iron accumulation. We investigated non-heme iron concentration, oxidative stress to nucleic acids in gastrocnemius muscle and key indices of sarcopenia (muscle mass and grip strength) in male Fischer 344 X Brown Norway rats fed *ad libitum* (AL) or a calorie restricted diet (60% of *ad libitum* food intake starting at 4 months of age) at 8, 18, 29 and 37 months of age. Total non-heme iron levels in the gastrocnemius muscle of AL rats increased progressively with age. Between 29 and 37 months of age, the non-heme iron concentration increased by approximately 200% in AL-fed rats. Most importantly, the levels of oxidized RNA in gastrocnemius muscle of AL rats were significantly increased as well. The striking age-associated increase in non-heme iron and oxidized RNA levels and decrease in sarcopenia indices were all attenuated in the calorie restriction (CR) rats. These findings strongly suggest that the age-related iron accumulation in muscle contributes to increased oxidative damage and sarcopenia, and that CR effectively attenuates these negative effects.

## Introduction

Aging is a process of deleterious and progressive changes in multiple organ systems. The consequences of the aging process in skeletal muscle are characterized by a decline in physiological function, reduced physical activity, and a loss of muscle mass, quality and strength, a condition often referred to as sarcopenia [1]–[3]. Evidence suggests that an acceleration in the production of reactive oxygen species (ROS) cause oxidative damage to a variety of tissue, including skeletal muscle, with aging and this may be a major contributor to sarcopenia [3]–[5]. Skeletal muscle cells continuously generate ROS, such as superoxide anion and hydrogen peroxide, which are diffusible within skeletal muscle cells derived from diatomic oxygen via several endogenous and exogenous pathways [3], [6], [7]. Superoxide anions and hydrogen peroxide may act as signaling molecules, altering cellular function [8]. In contrast, hydroxyl radicals may be formed via Fenton chemistry in the presence of redox active free iron or other transition metals and react immediately with any surrounding biomolecules [9]–[11]. Normally, the biological activity of ROS is kept in balance by specifically localized antioxidants and antioxidant enzymes in skeletal muscle. However, with aging, oxidant production in skeletal muscle may exceed the antioxidant capacity to buffer oxidants resulting in oxidative damage [6], [12], [13]. Oxidation of biomolecules can alter the structure and function of lipids, proteins, and nucleic acids, leading to cellular dysfunction and even cell death [7], [14]. However, the precise origin and sources of oxidative stress in skeletal muscle have not been determined.

Iron is recognized as a potent pro-oxidant and a necessary catalyst for the formation of reactive oxygen species in biological systems [15]. The release of iron from iron-binding proteins results in the formation of ROS [16], [17]. Increasingly, it is apparent that iron-associated oxidant damage is intimately involved with diseases of aging, from cancer to Alzheimer's [18]–[22]. However, the role of iron homeostasis with normal aging in tissues like muscle is rather unclear. Moreover, the significance of oxidant production by iron and its potential causal role in sarcopenia has not been fully investigated. Recently, Altun *et al*. used 2-dimensional gel electrophoresis and mass spectrometry to examine the changes in proteins in the gastrocnemius muscle of adult (4 months) and aged (30 months) male Sprague-Dawley rats [23]. They found that levels of muscle non-heme iron and the iron transport protein, transferrin, were elevated in senescence, suggesting that iron load is a significant component of sarcopenia [23].

In 1998, Cook and Yu reported that age-related iron accumulation in liver, brain and kidney was markedly suppressed by calorie restriction (CR), an intervention which has been recognized as an important model to better understand age-related chronic diseases in variety of species [24]–[28]. It was shown that CR attenuates the aging process by enhancing DNA repair potential [29], reducing metabolic rate [30], improving insulin sensitivity [31], and decreasing oxidant production [32]. Although the beneficial effects of CR on aging are well established, little is known about the effect of CR on age-related iron accumulation in skeletal muscle. Therefore, we investigated non-heme iron levels in skeletal muscle, oxidative stress and functional outcomes (grip strength and muscle mass) in *ad libitum*-fed or calorie-restricted rats. This study provides valuable insights into the possible causal role of iron as well as provides us with a potential target for intervention to prevent sarcopenia.

## Materials and Methods

### Animals and experimental procedures

The proposed study was approved by the Institutional Animal Care and Use Committee at University of Florida. All procedures were performed in accordance with the National Institutes of Health guidelines for the care and use of laboratory animals. *Ad libitum (*AL) fed and calorie restricted (CR) male Fischer 344 X Brown Norway rats were obtained from the National Institute of Aging colony (Harlan Sprague Dawley, Indianapolis, IN) at 8, 18, 29 and 37 months of age. The rats were housed individually in the National Institute of Aging in a temperature-controlled (20±2°C) and light-controlled environment (12-hour light/dark cycle) with food pellets (NIH31 pellets #7017 for AL rats and NIH31/NIA pellets #7109 for CR rats) and water available *ad libitum*. Calorie restriction (10% restriction) was started at 3.5 months of age, increased to 25% restriction at 3.75 months, and maintained at 40% restriction from 4 months onward and throughout the individual animal's life. Although the CR rats consumed about 33% less iron daily (NIH31/NIA pellets #7109) as compared to the AL rats (NIH31 pellets #7017), the body weight of CR rats was ∼30–45% lower than that of age-matched AL rats (**Table 1**). Therefore, the daily iron intake of CR rats, when normalized to body weight, was similar as that of AL rats. In addition, iron balance is tightly regulated at the site of absorption [33], and therefore only a given amount of iron will be absorbed into the circulation [34]. This is true when the iron content of the diet is relatively normal. Only when dietary iron concentrations are very high can the amount of iron in the diet overwhelm the strict regulation of dietary iron absorption. Body weights were measured weekly and health status was checked by a veterinarian daily. The rats were acclimated for 2 weeks after arrival. Grip strength measurements were determined after acclimation, followed by 2 weeks' break, and then the rats were sacrificed by decapitation. The liver and the gastrocnemius (predominately type I red fiber was removed) were dissected, weighed, frozen in liquid nitrogen, and stored in −80°C until analysis.

**Table 1:** Changes in body weights, muscle weights and liver weights in 8-month, 18-month, 29-month and 37-month old *ad libitum* (AL) and calorie restricted rats.

Age	8 months		18 months		29 months		37 months	
Diet	AL	CR	AL	CR	AL	CR	AL	CR
Body weights (g)	377±15^ c^	268±5^ a^	479±6 ^d^	307±3^ b^	538±7 ^e^	318±3 ^b^	486±10 ^d^	270±4 ^a^
MW/BW (g/100 g)	0.50±0.01 ^de^	0.58±0.01 ^f^	0.44±0.01 ^c^	0.53±0.01 ^e^	0.33±0.01 ^b^	0.47±0.01 ^cd^	0.15±0.01 ^a^	0.30±0.02 ^b^
LW/BW (g/100 g)	2.70±0.03 ^ab^	2.79±0.02^ abc^	2.78±0.06^ abc^	2.60±0.05 ^a^	2.78±0.06^ abc^	2.66±0.04 ^ab^	2.96±0.10 ^c^	2.89±0.07 ^bc^

Values are means±SEM (n = 9). ^a,b,c,d,e,f^Different letters indicate values are significantly different (*p*<0.05 by Tukey's Multiple Comparison Test). MW/BW = gastrocnemius muscle-to-body weight ratio. LW/BW = liver weight-to-body weight ratio.

### Non-heme iron assay

The gastrocnemius and liver iron content was measured as described by Rebouche *et al*. [35]. Tissues (∼50 mg) of liver or muscle (for muscle the fat and tendons were removed) were homogenized in water (1∶10 w∶v) using a glass-glass Duall homogenizer on slush ice. An equal amount of an iron releasing and protein precipitating solution (1 N HCl and 10% (v/v) trichloroacetic acid) was added to an aliquot (100 µl) of the homogenates, a blank (water), or the iron standards, and the samples were incubated at 95°C for 60 min. Following centrifugation (10,000×*g*, 10 min, room temperature) to remove heme-containing proteins, 40 µl of supernatant was mixed with an equal amount of sample blank solution (1.5 M sodium acetate and 0.1% (v/v) thioglycolic acid), and 80 µl of supernatant was mixed with an equal volume of chromogen solution (0.508 mM ferrozine, 1.5 M sodium acetate, 0.1% (v/v) thioglycolic acid). The samples were incubated at room temperature for 30 min for color development, and the absorbance was read at 562 nm in a quartz cuvette using a Beckman DU 640 spectrophotometer. A commercially available iron standard (High-Purity Standards, Charleston, SC) was diluted to 2, 4, 6, 8, 10 µg iron/ml in deionized water. After correction for sample blanks, the iron concentration was calibrated against the iron standard curve and calculated as µg iron per gram of wet tissue weight. Iron total amount in tissue was calculated by iron concentration and tissue weight of individual animal.

### Grip strength-behavioral/functional testing

Forelimb grip strength was measured using an automated grip strength meter (Columbus Instruments, Columbus, OH) [36], [37]. The experimenter grasped the rat by the tail and suspended it above a grip ring. After about 3 seconds, the animal was gently lowered toward the grip ring and allowed to grasp the ring with its forepaws. The experimenter then quickly lowered the body to a horizontal position and tugged the tail until its grasp of the ring was broken. The mean force in grams was determined with a computerized electronic pull strain gauge fitted directly to the grasping ring, and was divided by body mass. Average measurements from 3 successful trials were taken as the final outcome. Successful trials were defined as those in which the animal grasped the ring with both forepaws and pulled the ring without jerking.

### Measurement of RNA and DNA oxidation using HPLC-ECD

Tissue samples were randomized and total RNA and DNA oxidation levels of gastrocnemius muscles were analyzed using a novel HPLC-ECD method [38]. This procedure is based on high-salt nucleic acid release from proteins, followed by removal of proteins/fats by organic solvents at neutral pH, all in the presence of the metal chelator deferoxamine mesylate (DFOM) at 0°C. Briefly, muscle pieces were thawed, stripped for tendons on ice, weighed (∼180 mg), minced, and homogenized on slush ice using a glass-glass Duall homogenizer in 2 ml (1∶10 w∶v) buffer (3 M guanidine thiocyanate (GTC), 0.2% (w/v) *N*-lauroylsarcosinate, 20 mM Tris, pH 7.5) containing 10 mM freshly dissolved DFOM. After transferring the solution into phase-lock gel (PLG) tubes, an equal volume of phenol/chloroform/isoamyl alcohol (25∶24∶1, pH 6.7) was added and the samples were immediately vortexed, followed by a 10 min vortexing period at 0°C to completely release nucleic acids as previously described [38]. After centrifugation (4,500×*g*, 5 min, 0°C), the aqueous phase was transferred into a new PLG tube and extracted with an equal volume of chloroform/isoamyl alcohol (24∶1). The samples were hand-shaken, centrifuged, and the aqueous phase was collected and nucleic acids were precipitated by addition of an equal amount of isopropanol. After centrifugation (10,000×*g*, 10 min, 0°C), nucleic acids were washed with 70% (v/v) ethanol, dried, dissolved in DNase and RNase-free water containing 30 µM DFOM, and hydrolyzed using 4 U nuclease P_1_ and 5 U alkaline phosphatase in buffer (30 mM sodium acetate, 20 µM ZnCl_2_, pH 5.3) at 50°C for 60 min. After filtration, the samples were analyzed by high performance liquid chromatography coupled to electrochemical and UV detection (HPLC-ECD/UV).

### Blood sampling, hemoglobin concentration and hematocrit level

After decapitation, the trunk blood was collected using a funnel and split into equal amounts for plasma and serum preparation using 6 ml plastic sodium heparin vacutainer (Becton Dickinson; Franklin Lakes, NJ) and 8.5 ml clot activator serum collection tubes (Becton Dickinson; Franklin Lakes, NJ) respectively. The plasma tubes were immediately inverted (3–5 times) to prevent coagulation and put on ice for 10 min after which hemoglobin and hematocrit was measured in a single drop of blood using a HemoPoint® H2 instrument with a single-use microcuvette from Stanbio Laboratory (Boerne, TX).

### Statistical analysis

Results are expressed as means±SEM. Statistical analyses were performed using two-way ANOVA followed by Bonferroni's post-tests. Statistics were calculated using GraphPad Prism Version 4.0 (GraphPad Software, San Diego, CA). Pair-wise multiple comparisons were made by the Tukey's Test. *P* values<0.05 were considered significant.

## Results

### Body weight and muscle atrophy

Sarcopenia is characterized by decreased muscle mass, quality and strength, in addition to increased muscle fatigability. The body weights of AL rats increased between the different age cohorts after 8 months of age until 29 months after which it showed a typical age-associated decrease (10% decline from 29 to 37 months; **Table 1**). Body weights in all the CR age groups were similar in the 8-, 18-, 29-, and 37- month-old rats (∼300 g). Overall, the CR rats were much lighter than their age-matched counterparts (**Table 1**). The gastrocnemius muscle-to-body weight ratio (MW/BW, g/100 g) decreased progressively with age both for AL and CR rats (**Table 1**). However, the age-associated decreases in the CR rats were significantly ameliorated as compared with their age-matched controls (*p*<0.001). The MW/BW in the CR rats were higher by 16% at 8 months, 19% at 18 months, 40% at 29 months, and 105% at 37 months as compared to their age-matched controls, indicating that the CR effects were much more pronounced at advanced age (**Table 1**).

The ratio of liver weight to body weight (LW/BW) remained fairly constant within all AL and CR groups until 29 months, but increased slightly at 37 months of age. The changes in liver weights in all groups were proportional to the body weight changes, and therefore there were no significant differences in LW/BW between CR and age-matched control rats (**Table 1**). These data show that the effects of aging on skeletal muscle mass and body weight ratio were quite different from those in liver.

### Hematological parameters with age and calorie restriction

Two key hematological parameters, hemoglobin (Hb) concentration and hematocrit (Hct) level, were measured to indirectly provide additional insights into iron status. The levels of Hct and Hb were both decreased slightly with age in the AL rats, with the lowest levels found in the 37-month-old animals as compared to all other groups. Two-way ANOVA showed a significant effect of age (*p*<0.0001) and CR (*p*<0.001). Overall, this gives a general indication that the animals were not anemic in both AL and CR rats.

### Non-heme iron in gastrocnemius muscle

**Figure 1:**
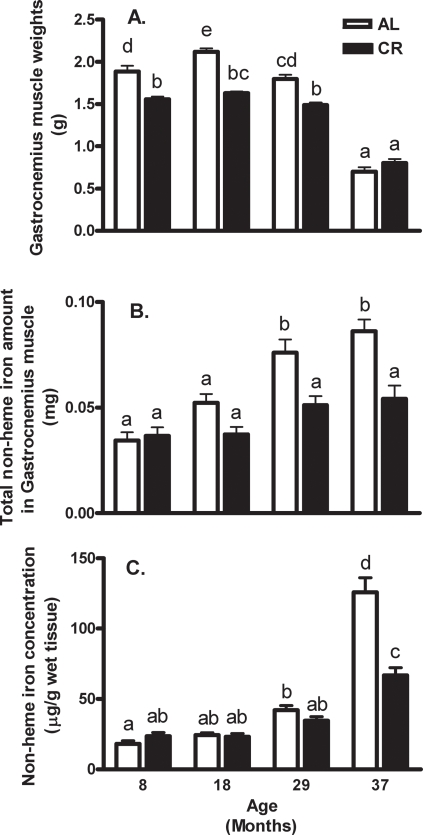
Effect of aging and calorie restriction on gastrocnemius weights and non-heme iron content. (A) Gastrocnemius muscle weights from *ad libitum*-fed and calorie-restricted rats at 8, 18, 29, and 37 months of age. (B) Total non-heme iron content of gastrocnemius muscle was calculated from non-heme iron concentrations and muscle weights. (C) Non-heme iron concentrations were measured colorimetrically after acid digestion of tissue. Values are means±SEM (n = 8–9). ^a,b,c,d^Different letters indicate values are significantly different (*p*<0.05 by Tukey's Multiple Comparison Test).

Wet muscle weights gradually declined with age in the AL rats, yet most rapidly between 29 and 37 months of age (**Fig. 1A**). The total amounts of non-heme iron increased significantly after 29 months of age in AL rats, whereas they did not change over time in CR rats (**Fig. 1B**). There was no significant change in non-heme iron concentration in the gastrocnemius muscle of both AL and CR rats between the ages of 8 and 18 months (**Fig. 1C**). At 37 months of age, non-heme iron concentrations increased by approximately 85% and 200% in the CR and AL rats, respectively, in comparison to 29-month- old rats. The increase in non-heme iron concentrations at 37 months is likely mostly due to muscle shrinkage (**Fig. 1A**). In addition, the non-heme iron concentrations in the 37-month-old AL rats were higher than in the CR rats because they had accumulated iron over time, whereas the CR rats did not (**Fig. 1B**). Although we reported both total non-heme iron (in mg) and intracellular iron concentration (in µg/g wet weight), the intracellular iron concentration is probably more relevant under pathological conditions in which iron binding and storage could become exceeded, releasing redox-active iron and increasing oxidative stress.

### Non-heme iron in liver

**Figure 2:**
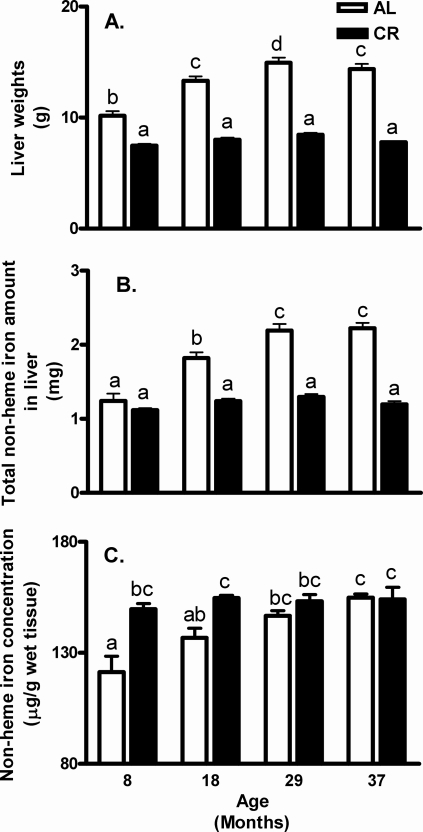
Effect of aging and calorie restriction on liver weights and non-heme iron content. (A) The liver weights increased with age in AL rats, but not in CR rats. (B) Total amount of non-heme iron in liver increased with age in AL rats, but not in CR rats. (C) Liver non-heme iron concentrations did not change over time in CR rats, but they increased in AL rats. Values are means±SEM (n = 9). ^a,b,c,d^Different letters indicate values are significantly different (*p*<0.05 by Tukey's Multiple Comparison Test).

The liver is the major iron storage organ and its iron levels accurately reflect total body iron stores [39]. Figure 2A-C show significant age-related iron accumulation in liver of AL rats only. Total liver non-heme iron amounts gradually increased with age in AL rats (**Fig. 2B**), but not in CR rats, which was attributed to the changes of liver weights and liver non-heme iron concentration (**Fig. 2A and 2C**). Two-way ANOVA showed the similar results for total liver iron amounts, iron concentration and liver weights, a significant age effect (*p*<0.0001), a significant CR effect (*p*<0.0001) and a significant interaction between age and diet (*p*<0.001). Overall, total non-heme iron amounts, non-heme iron concentration and liver weights in CR rats did not change over time. On the contrary, they increased significantly with age in AL rats.

### DNA/RNA oxidative damage in gastrocnemius muscle

**Figure 3:**
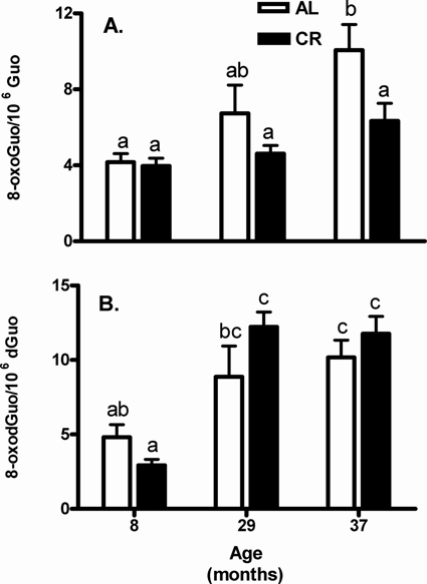
Effect of aging and calorie restriction on nucleic acid oxidation in gastrocnemius liver weights and non-heme iron content. A) Total RNA oxidative damage in the gastrocnemius muscles increased with age in AL rats, which did not change over time in CR rats. B) Total DNA oxidative damage in the gastrocnemius muscle increased markedly after 29 months of age in both AL and CR rats. Values are means±SEM (n = 6). ^a,b,c^Different letters indicate values are significantly different (*p*<0.05 by Tukey's Multiple Comparison Test).

Iron is a potent pro-oxidant and iron accumulation in aged skeletal muscle may increase oxidative stress to RNA and DNA as assessed by the levels of oxidative products 8-oxo-7,8-dihydroguanosine (RNA) and 8-oxo-7,8-2’-deoxyguanosine (DNA). We measured levels of oxidized RNA (**Fig. 3A**) and DNA (**Fig. 3B**) from AL and CR rats at 8, 29, and 37 months of age. RNA oxidative damage was significantly elevated (140%; *p*<0.01) in the gastrocnemius muscles of the 37-month-old AL rats as compared to 8-month-old AL rats (**Fig. 3A**) and two-way ANOVA revealed a significant age effect (*p*<0.05). The age-related RNA oxidative damage in 37-month-old AL rats was significantly attenuated by CR (*p*<0.05). The 29-month-old AL rats had a 60% increase in RNA oxidation over the 8-month-old AL rats, but this change was not statistically significant. DNA oxidative damage also increased with age in both the 37-month-old AL and CR rats (*p*<0.05) and two-way ANOVA indicated no CR effect on DNA oxidative damage (**Fig. 3B**).

### Grip strength (physical performance)

**Figure 4:**
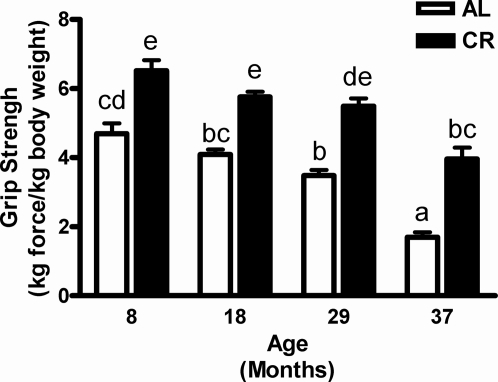
Effect of aging and calorie restriction on grip strength. Grip strength in CR rats declined significantly at 37 months of age, while it decreased significantly at 29 months of age in AL rats, yet more pronounced in advanced age of 37 months. Values are means±SEM (n = 7–8). ^a,b,c^Different letters indicate values are significantly different (*p*<0.05 by Tukey's Multiple Comparison Test).

Grip strength is an excellent indicator of muscle strength and it correlates significantly with sarcopenia and mortality [36], [37], [40]. In our study, there was a significant age-related decline for grip strength in the AL groups with a 13% decrease at 18 months (*p*>0.05), 26% decrease at 29 months (*p*<0.05) and 64% decrease at 37 months (*p*<0.001) as compared to 8-month-old rats, respectively (**Fig. 4**). The significant decrease of grip strength was already noticeable before 29 months of age in AL rats, whereas there was no significant decrease in grip strength in CR rats until 37 months of age. Most importantly, the CR rats at 37 months of age showed equivalent grip strength to 8-month-old AL rats. In general, the age-associated decline in grip strength was significantly attenuated by CR in all age groups (*p*<0.05), indicating that CR partly prevents the decline in physical performance with age (**Fig. 4**).

## Discussion

It has been well accepted that accumulation of iron in organs such as the liver and the heart is associated with cirrhosis and heart failure in genetic hemochromatosis and thalassemia patients [19], [41] and, in particular, high levels of iron in the brain have been strongly implicated to play a key role in the age-related decline in cognition [42]–[44]. However, limited data are available concerning the association between high levels of iron in skeletal muscle and sarcopenia. Recently, Altun *et al*. reported that the non-heme iron level in gastrocnemius muscle of aged male Sprague-Dawley rats was significantly elevated [23]. Since the skeletal muscle represents about 40% of body mass and contains 10% to 15% of body iron, which is mainly located in myoglobin and mitochondria [45], it is of great interest to improve our understanding of iron homeostasis in this tissue system. In agreement with Altun *et al*., our study shows that the iron accumulation may be a hallmark of aged skeletal muscle. The significant increase of non-heme iron amount in gastrocnemius muscle in AL rats was noticeable at 29 months of age, which concurred with a significant increase of oxidized nucleic acid damage. In particular, excessive oxidative stress is associated with the pathogenesis of sarcopenia [7]. Accordingly, we measured the gastrocnemius muscle mass and grip strength to advance our understanding of sarcopenia in the rats. In addition, a novel finding of this study is the remarkable attenuation of the age-associated rise of iron level in the gastrocnemius muscle with calorie restriction, paralleling a significant decrease of total RNA oxidative damage and a striking amelioration of muscle mass and grip strength.

Sohal *et al* have documented increased levels of redox-active iron (bleomycin-detectable iron assay) in aged liver, but in their study they did not provide a measure of oxidative stress [46]. Bleomycin assay, as a non-physiological iron chelator reaction, is developed to detect and measure reactive iron species in biological fluids. Sohal *et al's* study did not rule out the specific role of iron in modulation of molecular oxidative damage in certain tissues such as liver, where an age-associated increase in bleomycin-chelatable iron was observed. However, in other tissues such as the kidney, heart, and brain, no age-related elevation in the redox-active iron content was detected. Similarly, caloric restriction that lowers steady state amounts of oxidative damage did not always correspond to lower iron content. The very mixed pattern of findings would seem to be compatible with the view that either a) the bleomycin assay is unreliable in tissue homogenates as described by Galey [47] or b) the age-associated increase in molecular oxidative damage in various specific tissues may be caused by different combinations of factors that collectively lead to increases in the steady state levels of oxidative damage.

Increased iron levels have also been reported in kidney and brain in aged rats [15], [18], however, our current study shows the age-related increases of non-heme iron in livers are more modest than those observed in skeletal muscles. This may suggest that the iron accumulation in aged Fischer 344 X Brown Norway rats is tissue-specific, and iron accumulation in skeletal muscle may in part be related to the postmitotic nature of myofibers [4], [23]. Furthermore, a study from Okunade *et al*. has shown that the presence of significant levels of non-heme iron in the non-insulin-dependent diabetes mellitus subjects is an indication of the potential for iron-catalysed production of hydroxyl and other toxic radicals could cause continues oxidative stress and tissue damage [48]. Therefore, altered non-heme iron levels in aged rats may be a recognized age-related phenomenon which contributes to oxidative stress and muscle atrophy.

We detected increases in non-heme iron levels in liver of CR young rats, compared to AL rats (both in the 8- and 18-months) (**Fig. 2C** expressed as µg/g wet weight). The increase may be due to the fact that liver weight (**Table 1**) decreases acutely and rapidly following the initial 4-month calorie restriction intervention. For example, the differences between the 8-month AL liver weights (377±15 g) and calorie-restricted animals (268±5 g) is highly significant. It is also possible that iron import, storage and export in the liver is better maintained in all CR age groups. Note the very consistent similar level of total and normalized iron levels in all CR group ages (**Fig. 2C**).

The increases in non-heme iron levels in the young CR animals could have lead to more free radical damage. However, young CR animals i) are better protected against oxidative stress because of increased antioxidant defense systems [49] ii) CR animals may have greater levels of ferritin and frataxin proteins to store and transport iron and iii) non-heme iron may be tighter bound to proteins rendering them less redox active. It is also unknown what % of the non-heme iron is potentially redox active because we could not measure free iron. Redox free iron measurements using the bleomycin/iron-dependent DNA degradation assay in these samples is challenging. This method is used to indirectly quantify the labile iron pool and involves the measurement of bleomycin/iron-dependent DNA degradation or the direct assay of oxidized 2-deoxyribose in homogenized tissue samples [50]. The bleomycin assay for “free” iron was introduced by Gutteridge et al. in 1981 as a first attempt to detect and measure reactive iron species in biological fluids, and has since allowed the detection of several new clinical states of plasma iron overload. Moreover, the labile iron pool has been quantitated by several other techniques, including the use of DFO-chelatable iron-59 [51] and electron paramagnetic resonance spectroscopy [52]. It is, however, difficult to measure free iron concentration in tissue precisely. For example, the bleomycin molecule is a non-physiological iron chelator (Ka 10^15^) that forms an iron complex with non-biological redox properties, and this has led some to criticize the data obtained using the assay [53]. Most importantly, disrupting the cells by tissue homogenation alters the existing equilibrium between free and bound iron, as well as its oxidation state [47]. Therefore, the bleomycin assay is mostly used in biological fluids such as plasma to indirectly quantify the labile iron pool; in this study, the measurement of non-heme iron concentrations gives a much better overall status of the iron pool.

**Figure 5:**
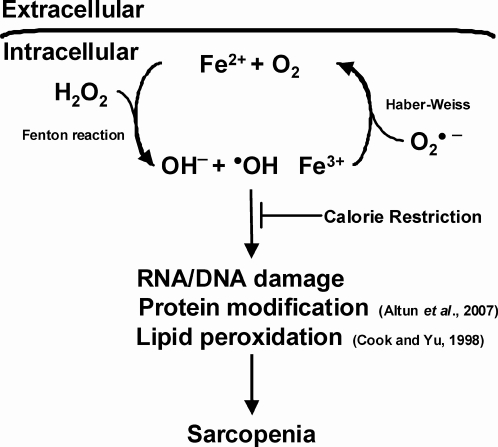
Calorie restriction attenuates iron accumulation and sarcopenia. Reactive iron plays a major role in oxidative stress via Fenton chemistry, where homolytic cleavage by ferrous iron (Fe^2+^) of H_2_O_2_ generating hydroxyl radicals. In this regard, both H_2_O_2_ and superoxide radicals have been shown to promote the release of iron from iron-binding proteins. Iron accumulation therefore increases the risk for RNA and DNA damage, protein modification, lipid peroxidation, and can affect protein synthesis which contribute to the process of sarcopenia.

We assessed oxidative stress to DNA guanine base as the oxidation product 8-oxo-7,8-dihydro-29-deoxyguanosine (8-oxodGuo) and found a significant age-related increase, in agreement with our previous study [38]. Cellular RNA oxidative damage product, 8-oxo-7,8-dihydroguanosine (8-oxoGuo), showed a similar age-associated increase and CR was able to attenuate the age-associated rise. Moreover, the strong association of iron accumulation with excessive ROS, which is also documented in other studies [15], [23], [54], suggest that decreases in non-heme iron levels and oxidized nucleic acid damage with CR may be more than coincidental (**Fig. 5**). Over the past decade, it is clear that iron, by virtue of its ability to both accept and donate electrons, contributes to excess production of damaging ROS through either Fenton or Haber-Weiss reactions [3], [55], [56]. ROS have been shown to mediate signaling pathways that regulate muscle atrophy (**Fig. 5**), especially in mammals [4], [9], [11], [57]. Hamilton *et al*. reported that the liver, heart and brain of the old C57BL/6 mice were more sensitive to the induction of oxo8dGuo in DNA by γ-irradiation because of enhanced levels of iron that potentiate the oxidative stress induced by γ-irradiation [58]. Rikans *et al*. also concluded that the increased sensitivity of hepatocytes from old rats to diquat was not because of changes in enzymatic mechanisms that protect against oxidative damage; rather, they found that hepatocytes from old rats were more sensitive to diquat because of higher levels of ferritin iron in the livers of the old rats [59]. In addition, a clinical study in a large sample of Japanese men and women demonstrated that the oxidative DNA damage measured by circulating 8-oxoGuo levels increased concurrently with serum ferritin levels in both sexes [60].

Moreover, data obtained herein show that RNA is more susceptible to oxidative stress than DNA, which is partially in agreement with our previous studies [38], [61]. The higher susceptibility of RNA could be due to a) its widespread cytosolic distribution where ROS and the iron is stored; b) repair or turnover mechanisms [38], [61]. Furthermore, RNA is single-stranded and may in fact have an enhanced potential for iron damage because it lacks protective histones [38]. In contrast, DNA is separated by the nuclear envelope and has less chance to interact with iron, which is mostly located in the cytosol. Oxidative damage to RNA can interfere with correct base pairing, which would compromise the accuracy of cellular processes such as translation [38]. The functional consequences of increased RNA oxidative damage in skeletal muscles could be serious and even causative to muscle atrophy [62]. Recently, oxidative damage to rRNA has been shown to result in decreased protein synthesis, which in skeletal muscles will eventually lead to muscle atrophy [63]. Therefore, the elevation in iron level of aged skeletal muscle, concomitant with increased total RNA and DNA oxidative damage, could play a major role to the observed muscle atrophy.

The increase of non-heme iron in the gastrocnemius muscle may also contribute to iron-mediated activation of apoptosis, resulting in muscle atrophy. Recent studies suggest that iron plays a key role in apoptosis induced by a variety of insults [64]–[66]. We also found (unpublished data) that decreased levels of cell death in aged gastrocnemius muscle are strongly associated with the reduced levels of non-heme iron by CR. Moreover, Carlini *et al*. reported that iron participated in the apoptosis stress pathway by up-regulating caspase-3 activity [64]. New evidence suggests that activation of caspase-3 is an initial step triggering accelerated muscle proteolysis. Specifically, caspase-3 activation promotes degradation of actomyosin complexes and leads to protein breakdown [3].

Grip strength is used as a marker of sarcopenia [36], [40]. Since, non-heme biochemical analyses are conducted on the gastrocnemius and strength is assessed using the forearm flexors, future studies need measure the amount of non-heme iron directly in the functional muscle assets to better reflect the relationship between iron accumulation and its impact on muscle strength. However, grip strength is used as an overall marker of sarcopenia and Carter *et al*. has shown that it is highly correlated with age in rats [36], [37], [40]. Compared to young AL rats, the decline of grip strength at 18 and 29 months of AL rats was 25% and 30%, respectively and then reaches 63% at 37 months. Similarly, muscle mass evaluated by the ratio of gastrocnemius muscle weight-to-body weight (MW/BW) was significantly decreased with age in AL rats. Taken together, these findings suggest that the deterioration of both muscle mass and muscle strength is severely accelerated in advancing age and is associated with excessive oxidative nucleic acids. In contrast, grip strength in CR rats was 26% higher at 8 months, 40% higher at 18 months, 51% higher at 29 months and 71% higher at 37 months as compared to the age-matched AL rats, suggesting the potential of CR intervention to mitigate the severity of ROS-mediated muscle loss and muscle function with age. Furthermore, the reduced levels of total RNA oxidative damage, which is concurred with a decreased non-heme iron levels in aged skeletal muscle in CR rats also implies the possible role of iron in production of damaging ROS (**Fig. 5**). In agreement with our study, Cook and Yu reported that the CR intervention can attenuate age-related iron accumulation as well as lipid peroxidation in kidney and brain in Fischer 344 rats at 24 months of age [15]. These data strongly support the notion that CR, by virtue of preventing age-related iron accumulation in muscle, may mitigate ROS-mediated sarcopenia.

It is usually believed that an inadequate energy intake may be an important contributor in the progression of sarcopenia in elderly individuals [67]; however, optimal nutritional conditions have not been established. On the contrary, the aged AL rats with 67% more calorie intake in our study have more oxidative damage and suffer from sarcopenia more severely than age-matched CR rats. This implies that oxidative stress may regulate proteolytic pathways leading to muscle atrophy, and that CR can significantly slow the progression of this pervasive problem. What is more, the differential response with aging between tissue weights of muscle (losses) and liver (increases) is partly explained by increases in fat depositions. Morphological changes in the hepatic sinusoid are largely responsible for the large increases in liver weight observed with old age. The changes include thickening and defenestration of the liver sinusoidal endothelial cell, sporadic deposition of collagen and basal lamina in the extracellular space of Disse, and increased numbers of fat engorged [68].

In conclusion, our findings complement and extend previous observations in several ways. First, we have rats that are considerably older (37 months), and we find that gastrocnemius muscle non-heme iron levels change dramatically in these rats (i.e., 600% higher than 8-month-old controls). Secondly, we studied rats of four ages (8, 18, 29, and 37 months), which allows us to document the progression of changes over time and to make correlations, such as that with RNA oxidation. Thirdly, our study is unique because it includes indices of sarcopenia and shows strong correlations with iron level changes. Importantly, our study is the first to show that caloric restriction attenuates this age-related iron accumulation in muscle, and mitigates oxidative stress and sarcopenia. Whether iron accumulation is a causative factor or merely a consequence of aging is still unclear. However, the strong association of iron levels with oxidized nucleic acid damage and the connection of oxidative damage with markers of sarcopenia warrant potential targeted interventions in an attempt to reduce iron levels and mitigate sarcopenia. Our study provides valuable insights into the mechanisms of the beneficial factors of CR on sarcopenia. Additional studies need to determine if and how specific iron-related proteins change and if interventions such as metal chelation can attenuate the levels of oxidative stress, apoptosis and/or mitochondrial dysfunction with age.
